# On the analysis of mortality risk factors for hospitalized COVID-19 patients: A data-driven study using the major Brazilian database

**DOI:** 10.1371/journal.pone.0248580

**Published:** 2021-03-18

**Authors:** Fernanda Sumika Hojo de Souza, Natália Satchiko Hojo-Souza, Ben Dêivide de Oliveira Batista, Cristiano Maciel da Silva, Daniel Ludovico Guidoni

**Affiliations:** 1 Department of Computer Science, Federal University of São João del-Rei, Sao Joao del-Rei, MG, Brazil; 2 Laboratory of Immunopathology, Oswaldo Cruz Foundation – Minas, Belo Horizonte, MG, Brazil; 3 Department of Statistics, Physics and Mathematics, Federal University of São João del-Rei, Sao Joao del-Rei, MG, Brazil; 4 Department of Technology, Federal University of São João del-Rei, Sao Joao del-Rei, MG, Brazil; National Institute for Infectious Diseases Lazzaro Spallanzani-IRCCS, ITALY

## Abstract

**Background:**

Brazil became the epicenter of the COVID-19 epidemic in a brief period of a few months after the first officially registered case. The knowledge of the epidemiological/clinical profile and the risk factors of Brazilian COVID-19 patients can assist in the decision making of physicians in the implementation of early and most appropriate measures for poor prognosis patients. However, these reports are missing. Here we present a comprehensive study that addresses this demand.

**Methods:**

This data-driven study was based on the Brazilian Ministry of Health Database (SIVEP-Gripe) regarding notified cases of hospitalized COVID-19 patients during the period from February 26th to August 10th, 2020. Demographic data, clinical symptoms, comorbidities and other additional information of patients were analyzed.

**Results:**

The hospitalization rate was higher for male gender (56.56%) and for older age patients of both sexes. Overall, the lethality rate was quite high (41.28%) among hospitalized patients, especially those over 60 years of age. Most prevalent symptoms were cough, dyspnoea, fever, low oxygen saturation and respiratory distress. Cardiac disease, diabetes, obesity, kidney disease, neurological disease, and pneumopathy were the most prevalent comorbidities. A high prevalence of hospitalized COVID-19 patients with cardiac disease (65.7%) and diabetes (53.55%) and with a high lethality rate of around 50% was observed. The intensive care unit (ICU) admission rate was 39.37% and of these 62.4% died. 24.4% of patients required invasive mechanical ventilation (IMV), with high mortality among them (82.98%). The main mortality risk predictors were older age and IMV requirement. In addition, socioeconomic conditions have been shown to significantly influence the disease outcome, regardless of age and comorbidities.

**Conclusion:**

Our study provides a comprehensive overview of the hospitalized Brazilian COVID-19 patients profile and the mortality risk factors. The analysis also evidenced that the disease outcome is influenced by multiple factors, as unequally affects different segments of population.

## Introduction

A new highly infectious-contagious coronavirus for humans emerged in December 2019 in Wuhan (Hubei province, China) [1, 2]. The initial outbreak in the Chinese region spread rapidly, but only on March 11, 2020 it was recognized as a pandemic by WHO [3]. The disease, called COVID-19, can cause severe pneumonia due to SARS-CoV-2 (severe acute respiratory syndrome coronavirus 2) infection. However, compared to other respiratory coronavirus syndromes (SARS-CoV and MERS-CoV), the lethality rate is low despite the high potential for spread [4].

The worldwide traffic of people facilitated the rapid spread of COVID-19, reaching countries on all continents in a short time. According to WHO data from February 9th, 2021, over 105 million cases of COVID-19 have been confirmed, including nearly 2.3 million deaths worldwide. Brazil has officially recorded the first case of COVID-19 on February 26th, 2020, and has become epicenter of the pandemic with over 9.4 million confirmed COVID-19 cases and nearly 232,000 deaths at this time [5].

COVID-19 has a broad spectrum of the disease, ranging from asymptomatic to extremely severe cases. According to WHO, the majority (about 80%) of COVID-19 patients is asymptomatic or mild, 15% are severe, requiring oxygen, and 5% are critical cases demanding mechanical ventilation [5]. However, recent studies suggest that asymptomatic individuals are 40 to 45% of the infected population [6] or even only 20% [7].

The symptomatic infection is characterized by fever, generalized weakness, dry cough, headache, dyspnoea, and myalgia, as well as leukopenia, lymphocytopenia, neutrophilia, high levels of C-reactive protein, D-dimer, lactate dehydrogenase and inflammatory cytokines [2, 8, 9] and loss of smell and taste in the initial stage of infection [10]. Although biomarkers such as lactate dehydrogenase, D-dimer and inflammatory cytokines, as well as chest computed tomography are good disease severity indicators, these tests are not routinely performed in most health centers, with restrict application in clinical practice, mainly in developing countries.

Symptomatic cases of COVID-19 may progress to recovery or a very severe condition, characterized by acute respiratory distress syndrome (ARDS), cytokine storm, blood coagulation dysfunction, acute cardiac and kidney injury, and multi-organ dysfunction, resulting in patient death [1, 11–13]. Age and comorbidities may influence the disease outcome. In fact, several studies have shown that the elderly and the presence of comorbidities such as cardiac disease, diabetes, chronic lung disease and obesity contribute to a more severe infection outcome [9, 14].

There is still not enough vaccine or therapeutic drugs for the specific treatment of COVID-19. Therefore, quarantine and social distancing are being the recommended measures in an attempt to reduce the infection rate in order to avoid overloading healthcare systems [15].

The growing number of COVID-19 cases requiring hospitalization and admission to the ICU can lead to the collapse of the health system. Thus, the screening of hospitalized patients with COVID-19 at risk of poor prognosis could improve the flow of care in hospitals, avoid overload and contribute to reducing the mortality rate.

Data from unidentified COVID-19 positive patients who have been hospitalized are available on the integrated health surveillance platform of the Brazilian Ministry of Health. However, the database, named Brazilian Severe Acute Respiratory Syndrome Database of the *Sistema de Informação de Vigilância Epidemiológica da Gripe*, SIVEP-Gripe (https://opendatasus.saude.gov.br/dataset/bd-srag-2020) including data from COVID-19, displays incomplete reports for demographic data, symptoms, comorbidities, ICU admission, etc.

We believe a comprehensive study on the characteristics and risk factors of Brazilian COVID-19 patients is missing. Thus, herein, we propose:

- Building a consistent dataset containing demographic data, clinical symptoms and comorbidities, ICU admission and use of ventilation of Brazilian hospitalized COVID-19 patients: those who were discharged and those who deceased, in order to investigate possible risk factors.
- Analyzing the mortality risk factors for different subgroups of hospitalized COVID-19 patients using the created dataset.

## Materials and methods

### Ethics statement

This retrospective study is based on a publicly available database and did not directly involve patients; it did not require approval by an ethics committee.

### Data extraction

**Fig 1:**
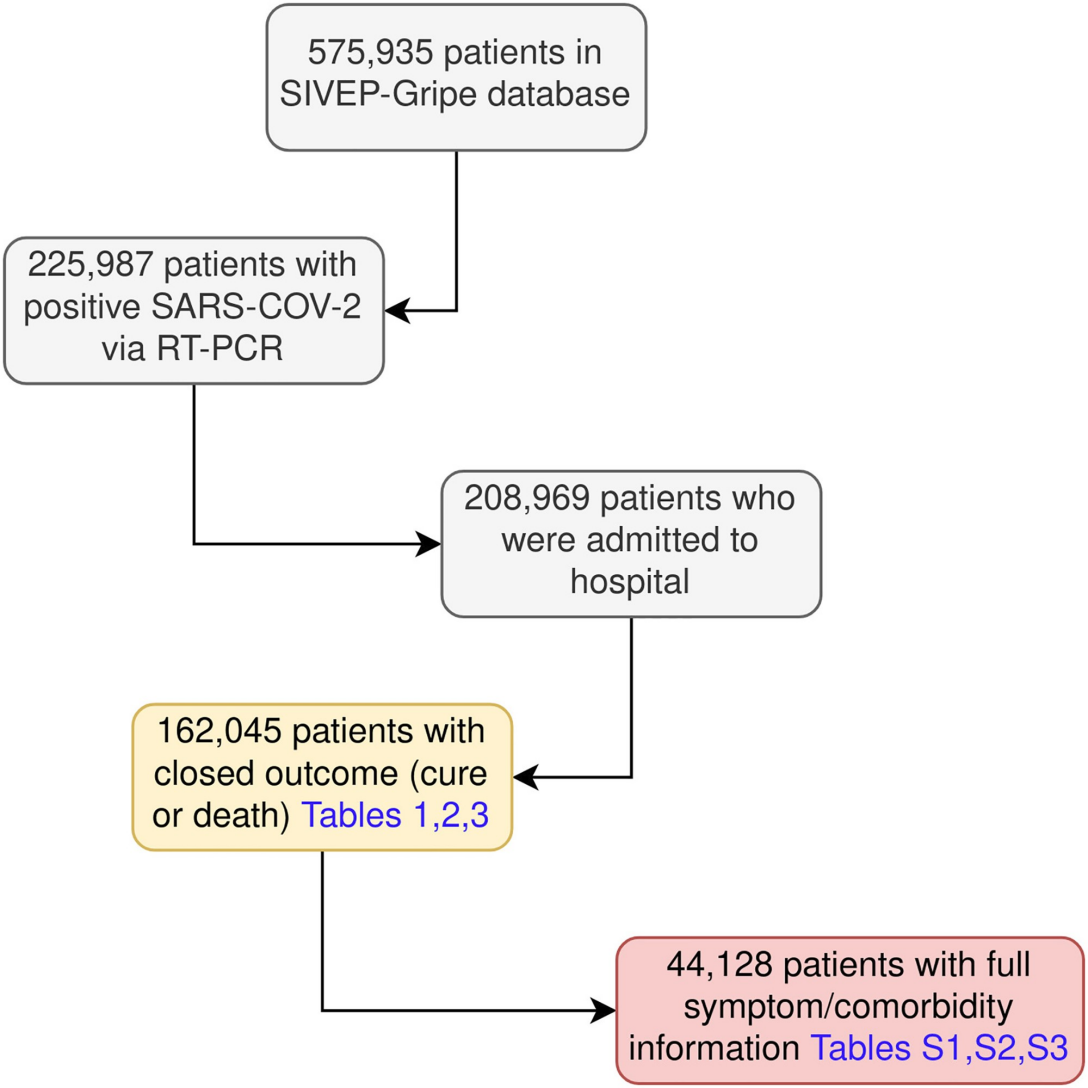
Flowchart of SIVEP-Gripe data used in the study.

In order to build the dataset to outline the profile of hospitalized COVID-19 patients, we downloaded the Brazilian Severe Acute Respiratory Syndrome Database, covering the time from February 26th to August 10th, 2020. The retrospective data used in this study was accessed on August 17th, 2020. This database contains individual information of patients hospitalized due to severe acute respiratory syndrome. The criteria for hospitalization according to the Ministry of Health concerns the individual presenting gripal syndrome along with dyspnoea/respiratory distress or persistent pressure in the chest or blood oxygen saturation <95% in room air or blue lips/face. The gripal syndrome concerns the individual with acute respiratory condition, characterized by at least two of the following signs and symptoms: fever (even if referred), chills, sore throat, headache, cough, runny nose, olfactory disorders or taste disorders. Laboratorial tests for confirmation of the SARS-CoV-2 are performed by molecular (RT-PCR) or immunological diagnostic (screening for antibodies or antigens), along with tests for detecting Influenza, Adenovirus, among others. The final diagnosis is registered as soon as the causer agent is identified. As shown in Fig 1, 225,987 patients were positive SARS-CoV-2 confirmed by RT-PCR method, with 208,969 patients being admitted to hospital according to information contained in SIVEP-Gripe Database. According to this data, the hospitalization rate of COVID-19 patients was 6.84% by August 10th, 2020.

### Dataset creation

We used in our study data from 162,045 patients who had closed outcome (cure or death) in order to provide a profile overview of the patients and after, a 44,128 patients cohort with full symptom/comorbidity information aiming to analyze risk factors for mortality.

Our dataset comprised information regarding gender, age range, region of residence, race/color and education. Symptoms such as fever, cough, sore throat, dyspnoea, respiratory distress, O2 saturation, diarrhea and vomiting were recorded. Comorbidities included cardiac disease, hematological disease, Down’s syndrome, liver disease, asthma, diabetes, neuropathy, pneumopathy, immunodepression, kidney disease and obesity. In addition, S1–11 Tables about Flu vaccine status, Flu antiviral usage, ICU admission and ventilation requirement have also been included. Temporal information considered the hospitalization and outcome dates.

### Variables and statistical analyses

In order to perform our statistical analysis, variables are classified accordingly: categorical nominal variables (region of residence, gender, race), categorical ordinal variables (age range, education), categorical binary variables (symptoms, comorbidities, Flu vaccine status, Flu antiviral usage, ICU admission, ventilation requirement) and continuous variables (time between hospitalization and outcome). Descriptive statistics are used to provide the features of the data in study. Categorical variables are given in absolute numbers and percentages. Continuous variables are reported through medians with IQRs (interquartile range). Lethality rate were estimated by gender, age range, region of residence, race/color and education.

Cox proportional hazards model [16] was used to investigate the association between the survival time of patients and the set of predictor variables. The outcome variable was time to death, defined as the time between hospitalization and outcome (for COVID-19 positive patients who died in hospital), with censoring on August 10th, 2020 for individuals who were alive by the end of the study period. The Hazard Ratio (HR) was measured to identify risk factors (the effect of a variable) on the outcome of interest. We estimate hazard ratios with 95% Confidence Interval (CI) in adjusted and unadjusted regression models analysis, since in most of situations several explanatory variables potentially affect the patient prognosis.

On the other hand, when fitting a model with many variables, it is often the case that some of them are in fact not associated to the event in study. Thus, selecting the set of variables to be considered is also an important task. Variables in the adjusted models were selected following the strategy proposed by Collett [16], consisting of a step-wise, iterative, optimizing algorithm. The algorithm minimizes $-2log\hat{L}$, where $\hat{L}$ stands for the maximum likelihood of a variable. We start fitting the model with each single variable, including those with *p* < 0.05. The model is then improved, following a sequence of steps which add/remove the variables aiming to achieve a less complex model, without noise and more easily interpreted.

All analyses were performed using Python (version 3.6.9) and the statistical package lifelines (version 0.24.16) [17]. *P* values <0.05 were considered statistically significant. For some of the variables, there was some indication of proportionality violation. However, according to a recent study, there is evidence that almost any clinical study will present some type of violation [18]. Results should be therefore interpreted as a weighted average of the true hazard ratios over the entire follow-up duration.

## Results

### Profile of hospitalized COVID-19 patients

Our study dataset included information of hospitalized COVID-19 patients, confirmed by RT-PCR. Demographic data of the study population were disaggregated into the cure and death subgroups (Table 1), showing an overall lethality rate of 41.28%. There was a predominance of cases in the Southeast region (58.96%) in the covered period. Moreover, the number of cases was higher among the elderly over 60 years (53.20%), male gender (56.56%) and, white (50.25%) and brown color patients (41.03%).

**Table 1:** Demographic data of the study population.

	all n(%)	cure n(%)	death n(%)	lr^a^(%)
Region	162045 (100.00)	95149 (58.72)	66896 (41.28)	
South	16088 (9.93)	10878 (11.43)	5210 (7.79)	32.38
Southeast	95541 (58.96)	60169 (63.24)	35372 (52.88)	37.02
Midwest	9928 (6.13)	6227 (6.54)	3701 (5.53)	37.28
Northeast	30114 (18.58)	13269 (13.95)	16845 (25.18)	55.94
North	10374 (6.40)	4606 (4.84)	5768 (8.62)	55.60
Age Range^b^				
0-4	1458 (0.90)	1265 (1.33)	193 (0.29)	13.24
5-9	348 (0.21)	311 (0.33)	37 (0.06)	10.63
10-19	999 (0.62)	837 (0.88)	162 (0.24)	16.22
20-29	5312 (3.28)	4598 (4.83)	714 (1.07)	13.44
30-39	15381 (9.49)	13127 (13.80)	2254 (3.37)	14.65
40-49	22952 (14.16)	18202 (19.13)	4750 (7.10)	20.70
50-59	29383 (18.13)	20312 (21.35)	9071 (13.56)	30.87
60-69	32935 (20.32)	17634 (18.53)	15301 (22.87)	46.46
70-79	28677 (17.70)	11648 (12.24)	17029 (25.46)	59.38
80-89	19159 (11.82)	5939 (6.24)	13220 (19.76)	69.00
90+	5441 (3.36)	1276 (1.34)	4165 (6.23)	76.55
Gender				
male	91656 (56.56)	52874 (55.57)	38782 (57.97)	42.31
female	70389 (43.44)	42275 (44.43)	28114 (42.03)	39.94
Race	110503 (100.00)	63173 (57.17)	47330 (42.83)	
white	55533 (50.25)	34145 (54.05)	21388 (45.19)	38.51
black	7683 (6.95)	4184 (6.62)	3499 (7.39)	45.54
asian	1659 (1.50)	924 (1.46)	735 (1.55)	44.30
brown	45334 (41.03)	23772 (37.63)	21562 (45.56)	47.56
indigenous	294 (0.27)	148 (0.23)	146 (0.31)	49.66
Education^c^	54547 (100.00)	33117 (60.71)	21430 (39.29)	
illiterate	3321 (6.09)	1156 (3.49)	2165 (10.10)	65.19
ES-1	13767 (25.24)	6560 (19.81)	7207 (33.63)	52.35
ES-2	10114 (18.54)	5705 (17.23)	4409 (20.57)	43.59
HS	17527 (32.13)	12042 (36.36)	5485 (25.59)	31.29
HE	8722 (15.99)	6710 (20.26)	2012 (9.39)	23.07
NA	1096 (2.01)	944 (2.85)	152 (0.71)	13.87

^a^lethality rate

^b^in years

^c^ES-1 = Elementary School 1; ES-2 = Elementary School 2; HS = High School; HE = Higher Education, NA = Not Applicable (age<7)

**Fig 2:**
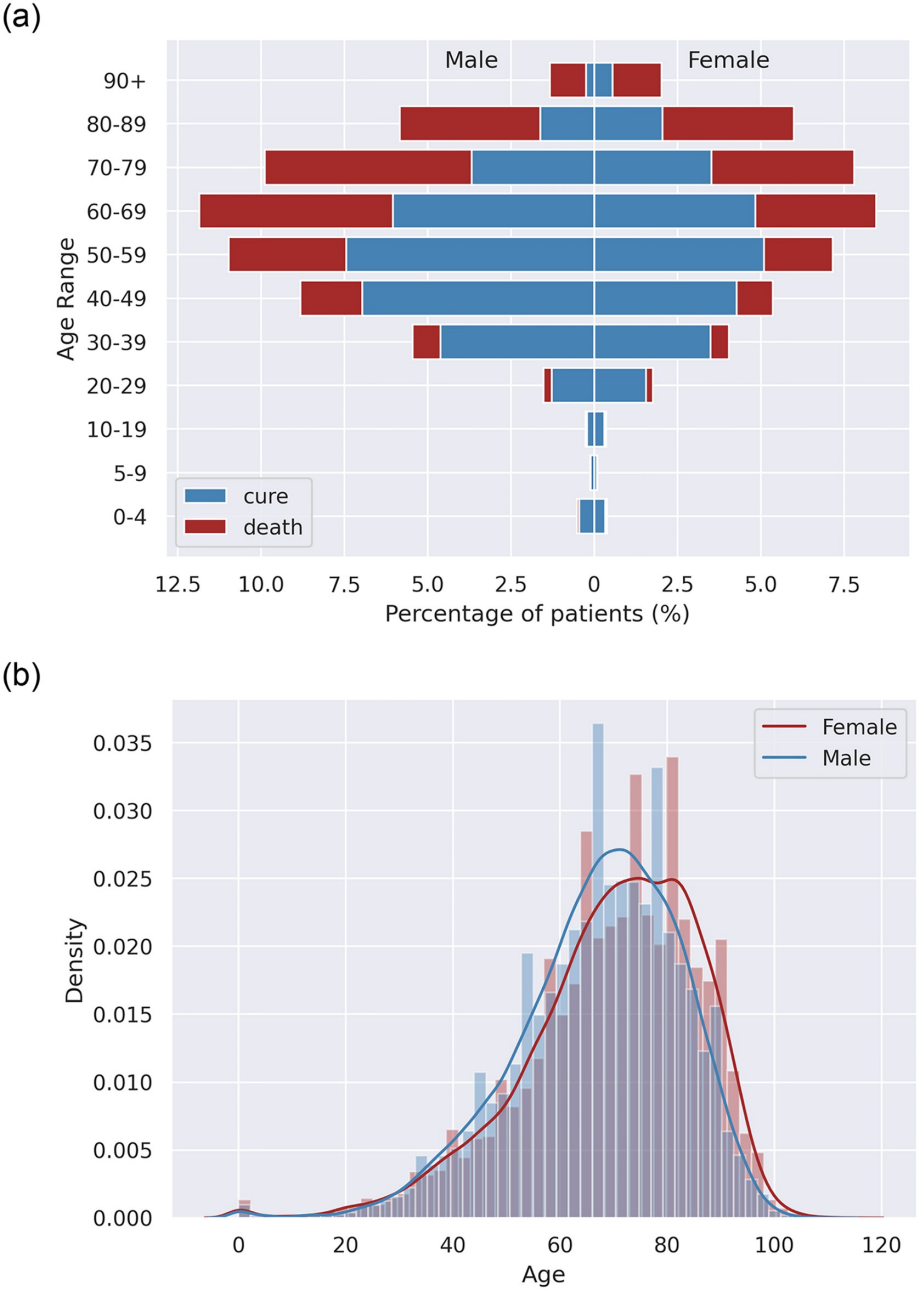
Outcome (A) and mortality density (B) distribution by age disaggregated data and gender of the study population (n = 162,045).

The distribution by sex and age group is shown in Fig 2. The lethality rate in hospitalized COVID-19 patients aged <20 years was very low, in accordance with observations made in other populations. The lethality rate was higher in the elderly of both sexes.

Besides a higher hospitalization rate in male gender (56.56%), the lethality rate was slightly higher in the male gender (Table 1). Moreover, age-disaggregated data (Fig 2) showed that the influence of gender on the risk of death is dependent on the age range.

**Fig 3:**
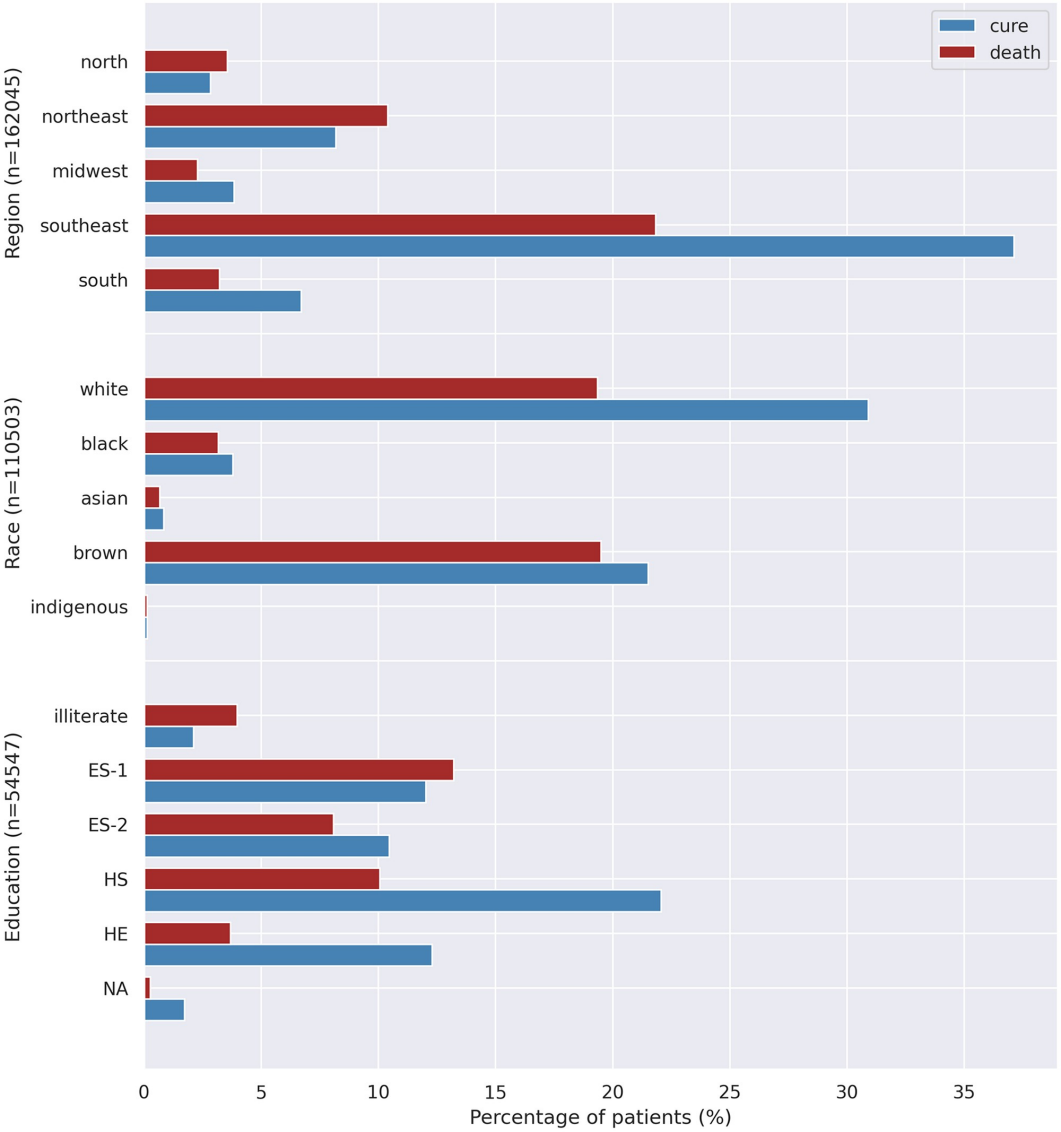
Outcome distribution according to demographic characteristics. Different number of patients apply (Brazilian regions: n = 162,045, race: n = 110,503, education: n = 54,547).

Lethality rate analysis showed a high rate in the North/Northeast region, in the highest age groups, in non-white populations and in the lowest educational levels (see Table 1 and Fig 3). The higher lethality in the North/Northeast region may be due to socioeconomic conditions and availability of beds in the ICU. On the other hand, it may also be due to the lack of appropriate knowledge about the characteristics of the new disease whose spread was highest in this region at the beginning of the pandemic.

**Fig 4:**
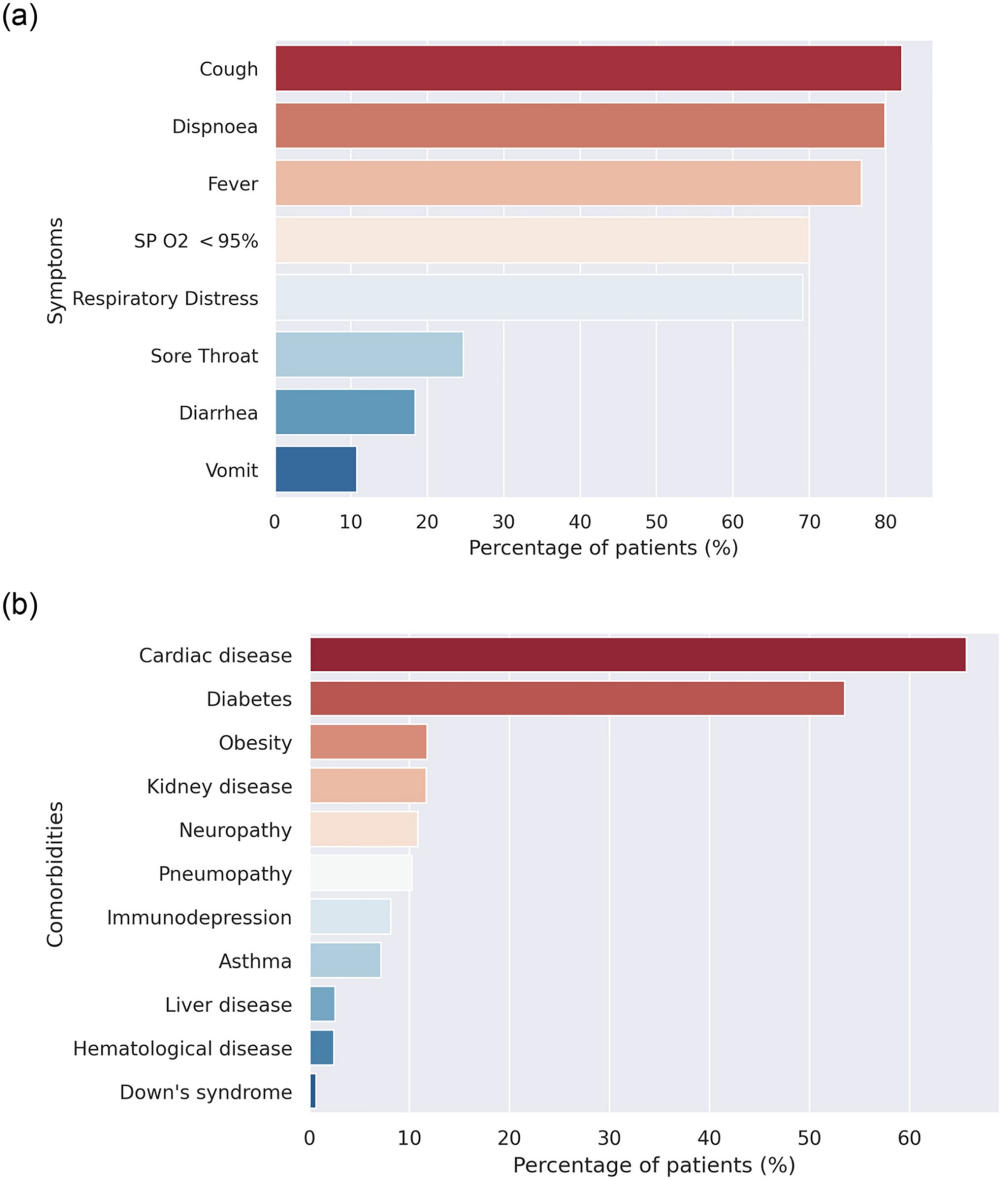
Most frequent clinical characteristics (A) and comorbidities (B) in the study population.

The most frequent symptoms and comorbidities in hospitalized COVID-19 patients are shown in Fig 4 and Table 2. Importantly, cough, dyspnoea, fever, low oxygen saturation and respiratory distress (Fig 4A), and cardiac disease, diabetes, obesity, kidney disease, neuropathy, and pneumopathy (Fig 4B) were more prevalent.

**Table 2:** Clinical data of the study population.

	all n(%)	cure n(%)	death n(%)
Symptom			
Fever (n = 145071)	111435 (76.81)	68783 (61.72)	42652 (38.28)
Cough (n = 146497)	120292 (82.11)	74154 (61.64)	46138 (38.36)
Sore Throat (n = 119158)	29420 (24.69)	19261 (65.47)	10159 (34.53)
Dispnoea (n = 145342)	116132 (79.90)	64024 (55.13)	52108 (44.87)
Respiratory Distress (n = 134430)	92991 (69.17)	49453 (53.18)	43538 (46.82)
SP O2 <95%^a^ (n = 135790)	95080 (70.02)	48772 (51.30)	46308 (48.70)
Diarrhea (n = 117100)	21536 (18.39)	14367 (66.71)	7169 (33.29)
Vomit (n = 114281)	12298 (10.76)	7953 (64.67)	4345 (35.33)
Other (n = 116778)	55974 (47.93)	37771 (67.48)	18203 (32.52)
Comorbidity			
Cardiac disease (n = 86244)	56664 (65.70)	28302 (49.95)	28362 (50.05)
Hematological disease (n = 63015)	1544 (2.45)	725 (46.96)	819 (53.04)
Down’s syndrome (n = 62832)	433 (0.69)	211 (48.73)	222 (51.27)
Liver disease (n = 62711)	1613 (2.57)	635 (39.37)	978 (60.63)
Asthma (n = 63873)	4566 (7.15)	3047 (66.73)	1519 (33.27)
Diabetes (n = 80155)	42919 (53.55)	20824 (48.52)	22095 (51.48)
Neuropathy (n = 65159)	7075 (10.86)	2598 (36.72)	4477 (63.28)
Pneumopathy (n = 64721)	6627 (10.24)	2603 (39.28)	4024 (60.72)
Immunodepression (n = 63844)	5195 (8.14)	2370 (45.62)	2825 (54.38)
Kidney disease (n = 64884)	7601 (11.71)	2693 (35.43)	4908 (64.57)
Obesity (n = 63022)	7413 (11.76)	4196 (56.60)	3217 (43.40)
Other (n = 77316)	45849 (59.30)	23199 (50.60)	22650 (49.40)

^a^blood oxygen saturation

Additional information from hospitalized COVID-19 patients is shown in Table 3. The ICU admission rate was 39.37% (of n = 145,053), with 62.40% death. In addition, 24.41% (of n = 138,962) required invasive mechanical ventilation, and of these 82.98% died. In contrast, only 31.83% died among those who required non-invasive ventilation. At least 72% (of n = 138,962) required some kind of ventilation, indicating that hospitalized patients can lead to the collapse of healthcare systems. The mortality rate in the Influenza vaccinated group (33.64% of n = 62.695) and in the Influenza antiviral group (37.92% of n = 124,844) were 37.82% and 39.80%, respectively.

**Table 3:** Additional information of the study population.

	all n(%)	cure n(%)	death n(%)
Flu Vaccine (n = 62695)	21092 (33.64)	13116 (62.18)	7976 (37.82)
Flu Antiviral (n = 124844)	47344 (37.92)	28501 (60.20)	18843 (39.80)
ICU admission (n = 145053)	57103 (39.37)	21469 (37.60)	35634 (62.40)
Ventilation (n = 138962)	100268 (72.15)	51006 (50.87)	49262 (49.13)
IMV^a^ (n = 138962)	33916 (24.41)	5771 (17.02)	28145 (82.98)
NIV^b^ (n = 138962)	66352 (47.75)	45235 (68.17)	21117 (31.83)

^a^Invasive Mechanical Ventilation

^b^Non Invasive Ventilation

### Risk factors for mortality

Cox regression model was adapted for adjusted and unadjusted regression analyses, considering the time between hospitalization and outcome (median = 8 days, IQR 4-14). Such analyzes were based only on patients with complete data (n = 44,128) for symptoms, comorbidities, information regarding ICU admission, ventilation and temporal data, besides age and gender (S1–S3 Tables). Vaccine and antiviral information were considered negative in the absence of data. The general characteristics of this subgroup are representative of the study population presented in Tables 1–3.

**Fig 5:**
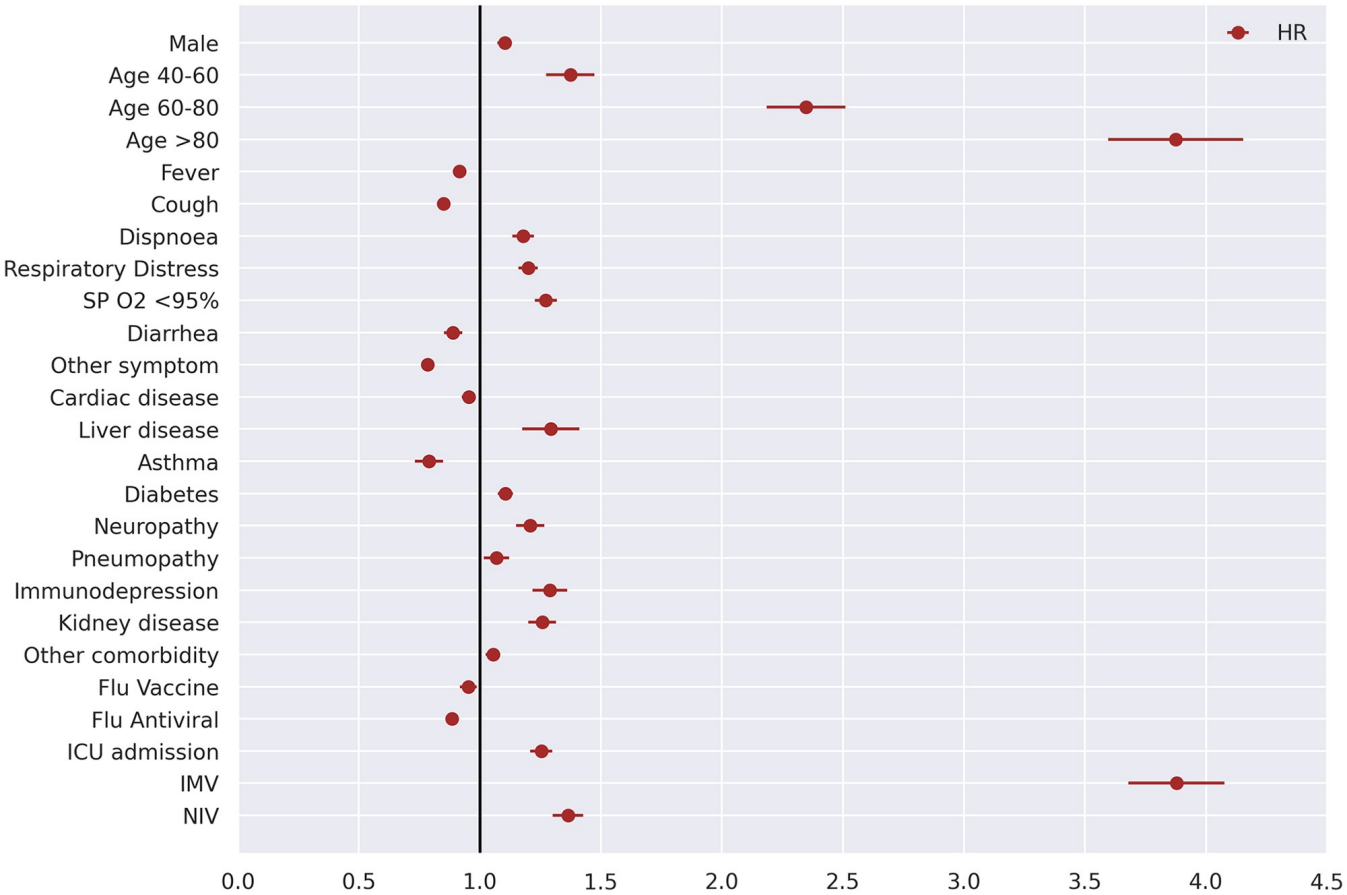
Mortality prognosis based on the general characteristics of patients in an adjusted Cox regression model (CI 95%) n = 44,128.

Although the lethality rate is ≈3.0% for notified COVID-19 cases in Brazil, the condition is very critical among hospitalized patients, with a ≈41% lethality according to Table 1. Mortality HRs for hospitalized COVID-19 patients are shown in Fig 5.

The unadjusted Cox regression analysis presented 27 variables (out of 28) as potential increase/decrease risk factors for mortality (S4 Table). Age categories count as one variable. However, after applying the variable selection strategy [16] for an adjusted regression model, 23 variables remained statistically significant in the adjusted regression analysis (Table 4), with a model concordance index of 0.74.

**Table 4:** Risk factors in fatal outcome using an adjusted Cox regression model (95% CI).

Variable^a^	HR	CI 95%	*p* value
Male	1.10	(1.07-1.13)	<0.001
Age 40-60	1.37	(1.27-1.48)	<0.001
Age 60-80	2.35	(2.19-2.52)	<0.001
Age >80	3.87	(3.60-4.17)	<0.001
Fever	0.92	(0.89-0.94)	<0.001
Cough	0.85	(0.82-0.88)	<0.001
Dispnoea	1.18	(1.13-1.23)	<0.001
Respiratory Distress	1.20	(1.16-1.24)	<0.001
SP O2 <95%	1.27	(1.23-1.32)	<0.001
Diarrhea	0.89	(0.85-0.93)	<0.001
Other symptom	0.78	(0.76-0.81)	<0.001
Cardiac disease	0.95	(0.93-0.98)	<0.005
Liver disease	1.29	(1.17-1.42)	<0.001
Asthma	0.79	(0.73-0.85)	<0.001
Diabetes	1.11	(1.07-1.14)	<0.001
Neuropathy	1.21	(1.15-1.27)	<0.001
Pneumopathy	1.07	(1.02-1.12)	0.010
Immunodepression	1.29	(1.22-1.37)	<0.001
Kidney disease	1.26	(1.20-1.32)	<0.001
Other comorbidity	1.05	(1.02-1.09)	<0.001
Flu Vaccine	0.95	(0.92-0.99)	0.009
Flu Antiviral	0.88	(0.86-0.91)	<0.001
ICU admission	1.25	(1.21-1.30)	<0.001
IMV	3.88	(3.68-4.09)	<0.001
NIV	1.36	(1.30-1.43)	<0.001

^a^see S2 Table; n = 44,128 patients with full symptom/comorbidity information

According to the adjusted Cox regression analysis, older age: 40-60 years old (HR = 1.37; 95% CI, 1.27-1.48), 60-80 years old (HR = 2.35; 95% CI, 2.19-2.52), >80 years old (HR = 3.87; 95% CI, 3.60-4.17) and invasive mechanical ventilation (HR = 3.88; 95% CI, 3.68-4.09) were the main risk factors for mortality. Besides that, noninvasive ventilation (HR = 1.36; 95% CI, 1.30-1.43), liver disease (HR = 1.29; 95% CI, 1.17-1.42), immunodepression (HR = 1.29; 95% CI, 1.22-1.37), low oxygen saturation (HR = 1.27; 95% CI, 1.23-1.32), kidney disease (HR = 1.26; 95% CI, 1.20-1.32), ICU admission (HR = 1.25; 95% CI, 1.21-1.30), neuropathy (HR = 1.21; 95% CI, 1.15-1.27), respiratory distress (HR = 1.20; 95% CI, 1.16-1.24), dyspnoea (HR = 1.18; 95% CI, 1.13-1.23), diabetes (HR = 1.11; 95% CI, 1.07-1.14), male gender (HR = 1.10; 95% CI, 1.07-1.13) and other comorbidity (HR = 1.05; 95% CI, 1.02-1.09) were also significantly associated with poor prognosis (Fig 5, Table 4).

Importantly, asthma comorbidity was not a mortality risk factor (HR = 0.79; 95% CI, 0.73-0.85). In addition, the use of Influenza antivirals (oseltamivir or zanamivir) showed a small reduction in the mortality risk (HR = 0.88; 95% CI, 0.86-0.91), as well as the Influenza vaccine (HR = 0.95; 95% CI, 0.92-0.99), according to the adjusted Cox regression analysis (Fig 5, Table 4).

### Risk factors for mortality by subgroups

**Fig 6:**
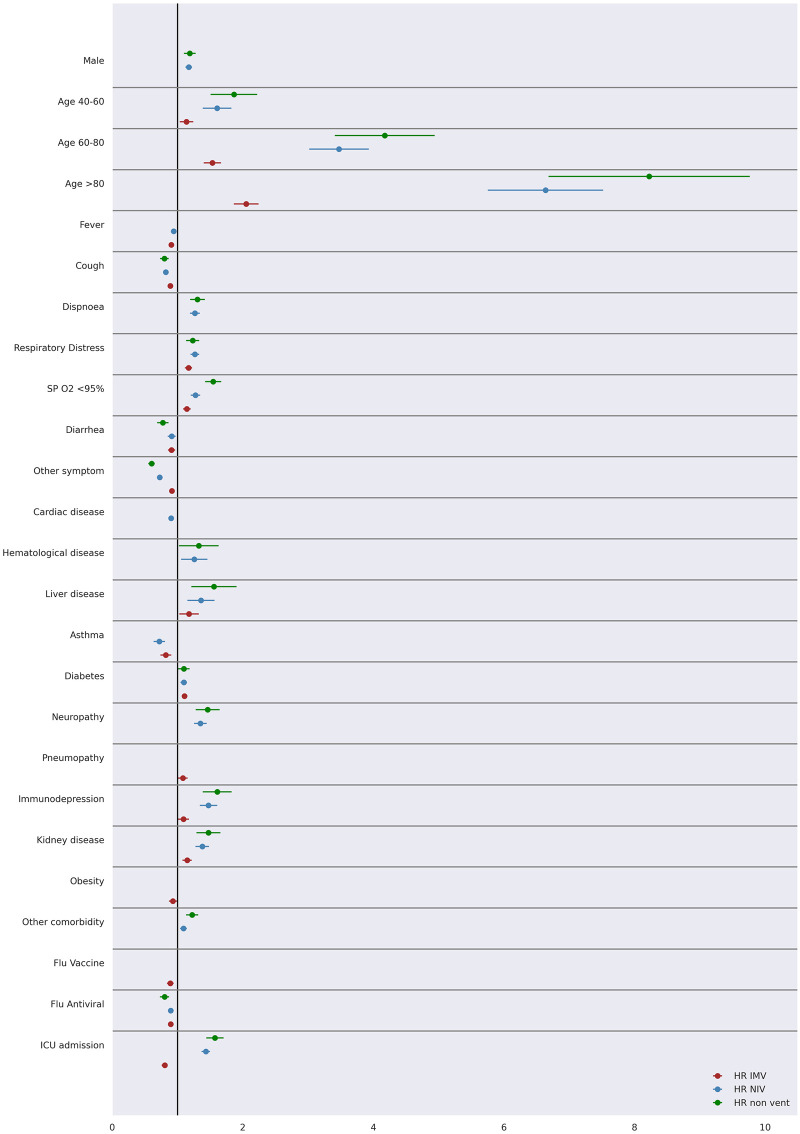
Mortality prognosis according to patient ventilation requirement in an adjusted Cox regression model (95% CI).

**Fig 7:**
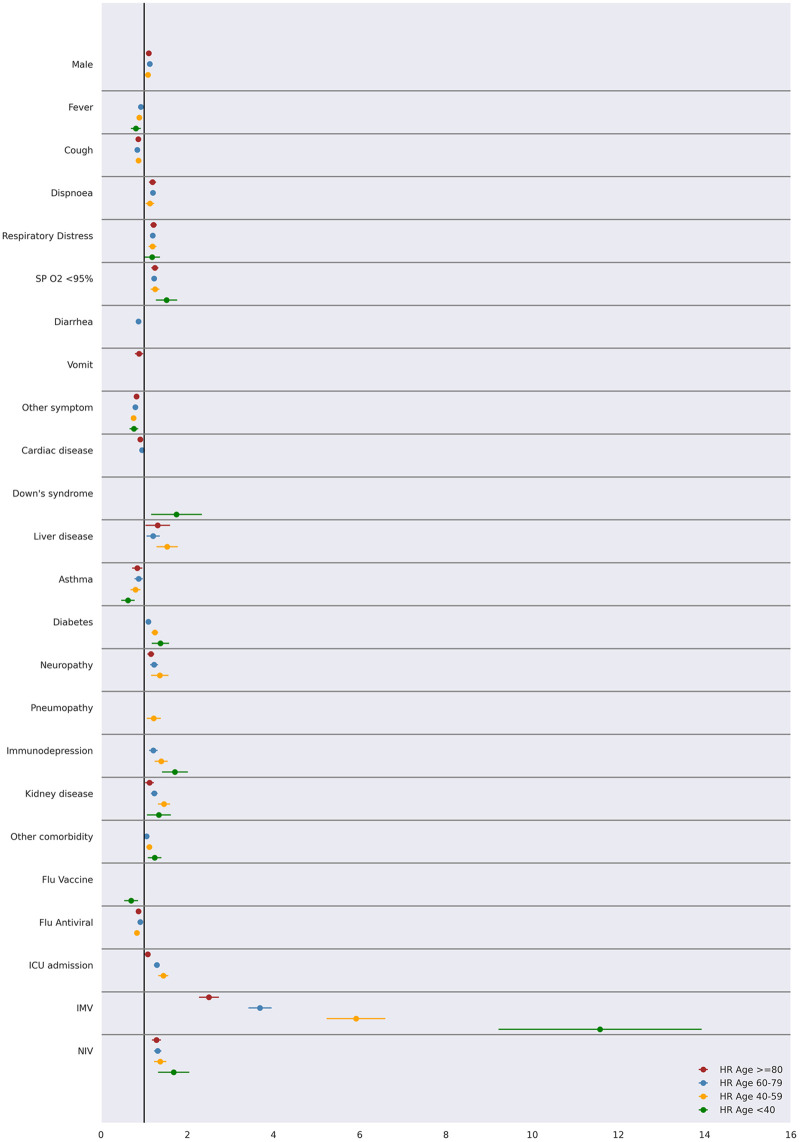
Mortality prognosis according to patient age range in an adjusted Cox regression model (95% CI).

Hospitalized COVID-19 patients data was disaggregated according to ventilation requirement and age range, whose hazard ratios are shown in Figs 6 and 7, respectively (all data referring to such figures are presented in S5–S11 Tables). In this case, an adjusted Cox regression model was fitted for each subgroup, following the same variable selection strategy described before. Therefore, a different set of variables showed significant in each subgroup.

According to Fig 6, older age is a risk factor independent of ventilation requirement or not. ICU admission, liver disease, immunodepression, kidney disease, neuropathy and hematological disease were the most critical risk factors for NIV and non-ventilation patients. In addition, clinical symptoms related to respiratory function and male gender were significantly associated with increased risk of mortality among NIV and non-ventilation patients. Diabetes indicated a slight increase in the risk of mortality for all patients. Influenza vaccine showed a small reduction in the risk of mortality only for patients that required IMV. On the other hand, the data suggest a small reduction in the risk of mortality for patients treated with Influenza antiviral regardless of the requirement for invasive ventilation.

The analysis of mortality risk factors for different age subgroups unveiled interesting results (Fig 7). Respiratory distress and low oxygen saturation were a risk factor for all ages and dyspnoea for patients over 60 years old. Ventilation requirements (IMV or NIV) are mortality risk factors for all ages, but it should be noted that IMV is a relevant risk factor especially for patients <40 years.

Importantly, diabetes (HR = 1.38; 95% CI, 1.17-1.61), Down’s syndrome (HR = 1.75; 95% CI, 1.16-2.64) and immunodepression (HR = 1.71; 95% CI, 1.42-2.07) are also mortality risk factors especially for younger patients (<40 years). The disaggregation by age range showed an interesting result for the Influenza vaccine. There was a reduction in the mortality risk for vaccinated patients aged <40 years (HR = 0.70; 95% CI, 0.54-0.91). On the other hand, Influenza antiviral showed a reduced risk of mortality for patients >40 years.

In synthesis, hospitalized COVID-19 patients <40 years who are diabetic, immunodepressed or requiring ventilatory support (IMV or NIV) are more likely to develop severe disease and die. On the other hand, older patients, male gender, presenting problems related to respiratory function and comorbidities such as liver disease, diabetes, neuropathy, immunodepression, kidney disease and requiring ICU and ventilation are at higher mortality risk.

## Discussion

Using the major Brazilian database for COVID-19 cases registration, we built a comprehensive dataset containing demographic data, clinical characteristics and comorbidities of hospitalized patients in order to investigate the epidemiological and clinical profile of the study population and the main mortality risk factors. The analysis of mortality risk factors is relevant to define criteria for classifying the severity of the disease and to provide better care to those who may need ICU admission and ventilatory support.

The profile of Brazilian hospitalized patients who deceased is similar to hospitalized U.S. patients who died, whose characteristics were older age, male gender and comorbidities such as immunodepression, kidney disease, chronic lung disease, cardiovascular disease, neurologic disorders, and diabetes [19].

A meta-analysis including COVID-19 patients from different countries showed that age is a determining factor on mortality, especially above 60 years of age [20]. Although susceptibility to infection may be similar in different age ranges, studies suggest that susceptibility to symptomatic infection increases with age, possibly related to immunosenescence and unregulated immune response [21]. A recent study showed that although the fatality rate is higher in the elderly population, the lethality associated with hypertension and diabetes comorbidities is more pronounced in younger people [22]. Here, we also observed that younger brazilian patients with diabetes and immunosuppression comorbidities are also more vulnerable to COVID-19.

In addition to older age, we found that male gender is an independent risk factor for mortality in COVID-19 patients. Our data are in line with a large study that covered data from 23 European countries, which showed that the mortality risk of COVID-19 patients is significantly higher in male than in female, regardless of socioeconomic characteristics and health systems in these countries [23]. A study conducted with hospitalized COVID-19 patients using the same Brazilian database showed that the mortality in the North region is higher, and in Pardo and Black patients, at least partly due to non-ICU admission [24]. Our data demonstrated that the lethality rate is higher in the North/Northeast region and among brown color patients being in agreement with this study.

A study using the same database (SIVEP-gripe) covering the period from January 1st, 2020 to June 8th, 2020 found that COVID-19 patients who received a recent Influenza vaccine (n = 36,650) had 8% less chance to need intensive care, 18% less chance of requiring invasive ventilation and 17% less chance of death [25]. However, in our adjusted Cox regression analysis (n = 62,695 with vaccine status), we found a slight association between Influenza vaccination and reduced risk of death, except for specific subgroups: patients under 40 years old and patients with IMV requirement. These differences may be due to our data extension (February 26th to August 10th, 2020), a period when the majority of the population had been vaccinated in the 2020 campaign. Studies on Influenza vaccination and possible protection against COVID-19 are controversial and present little evidence of a potential benefit. As suggested, the administration of the Influenza vaccine can be beneficial in preventing seasonal Influenza, avoiding super-infection or co-infections with COVID-19 [26].

The rate of hospitalized COVID-19 patients with obesity was 11.76%, of whom 43.40% died (Table 2). The adjusted regression analysis for obesity showed no significant association with mortality risk. Some studies have analyzed the association of obesity with poor prognosis for COVID-19 patients. Obese patients are at increased risk of developing severe illness [14]. The study presented by [27] showed a significant hazard ratio only for BMI ≥ 40Kg/m^2^ and, in another study, it was shown that BMI ≥ 35 kg/m^2^, in addition to increasing age and male gender, were independently associated with higher in-hospital mortality [28]. On the other hand, BMI >40 kg/m^2^, male gender and <60 years old were associated with higher mortality risk [29]. In addition to being a risk factor for severe COVID-19, high BMI was significantly associated with IMV requirement [30]. However, the Brazilian database does not discriminate overweight categories and BMI data is present for a few patients, so we were unable to perform categorized analysis.

The characterization of the profile of Brazilian patients admitted to ICU and requiring ventilation is relevant, as it can contribute to better care and mortality reduction. A recent review and meta-analysis involving COVID-19 patient data from several countries showed an ICU admission rate of 32%. The prevalence of mortality in the ICU was 39%, whereas in China this rate was higher, 42% [31]. Our analysis indicated that the events are more critical in Brazil, with 39.37% ICU admission and 62.40% lethality rate (Table 3), which may be related to infrastructure in public and private hospitals. The number of exclusive ICU beds for COVID-19 patients by June, 2020 was 9.43/100,000 population in Brazil, with 4.17 and 5.26 in the public and private health systems, respectively [32].

In accordance with some studies [27, 33], our data show that the invasive mechanical ventilation requirement among hospitalized COVID-19 patients is significant (24.41%), and with an extremely high lethality rate (82.98%) (Table 3). These data may be due to the high percentage of severely ill-hospitalized COVID-19 patients.

A meta-analysis study compiling data from several countries showed broad variability in ICU admission, IMV requirement and IMV lethality rates [34]. Brazilian COVID-19 patients ICU admission rate (39.37%) was similar to USA patients (35%). However, ICU mortality was extremely high (62.40%) compared to UK (33%), USA (29%), Italy (26%), China (24%), Spain (23%), France (15%), and Mexico (2%). Likewise, IMV patients lethality rate (82.98%) was higher than China (59%), UK (53%), USA (24%) and Mexico (4%). These high rates may be due to late admission to the ICU and delay in the mechanical ventilation introduction, or even the need to change the care protocol.

A recent study showed that asthmatic patients are not at risk and do not develop severe SARS-CoV-2 pneumonia compared to non-asthmatic patients [35]. In fact, we observed a low prevalence of asthmatic individuals (7.15%) among hospitalized COVID-19 patients, in agreement with the existing reports. Of note, the rate of cured asthmatics was twice that of those who died (Table 2) and asthma did not appear as a mortality risk factor (HR = 0.79; 95% CI, 0.73-0.85, p<0.001). It is speculated that type 2 immune response and therapeutic drugs used for asthma may have a potential protective effect [36].

A study of hospitalized patients showed that impaired renal function, elevated C-reactive protein and advanced age were the main predictors of in-hospital death [37]. Here, we also found that older patients and those with kidney disease are more susceptible to an unfavorable outcome.

Highly prevalent comorbidities among hospitalized COVID-19 patients were cardiac disease and diabetes, which is in line with observations made in other populations [8, 12]. Although the rate of hospitalized COVID-19 patients with heart disease was high (>50%), this comorbidity was not a critical mortality risk factor (HR = 0.95; 95% CI, 0.93−0.98). These data are in agreement with a study carried out in the Italian population [37]. In our previous cohort study on outpatients from a Brazilian state (ES) [38], we found a prevalence of 18.55% for cardiac disease and 7.89% for diabetes. Lethality rates in this cohort were 50.05% and 31.82% for cardiac and diabetic patients, respectively. Here, the in-patient study showed a prevalence of 65.7% and 53.55% for cardiac disease and diabetes comorbidities, respectively (Table 2). Lethality rates for patients with such comorbidities are extremely high, around 50%. Therefore, these observations indicate that patients with cardiac disease and diabetes are subgroups that deserve special attention.

Finally, it is worth to mention that in addition to the number of COVID-19 cases and differential tests between the new disease and endemic diseases, there is a concern regarding the continuity of disease eradication programs such as malaria [39]. Currently, COVID-19 has had a strong impact on the Brazilian Amazon, an endemic region for malaria. However, all efforts are focused on the care of COVID-19 patients. Certainly, new problems related to tropical diseases are expected to emerge in the future.

The main strength of this study is its scope, as it involved data from more than 162,000 hospitalized COVID-19 patients obtained from the major Brazilian database. Thus, it was possible to outline the demographic and clinical profile of hospitalized COVID-19 patients who were cured and those who deceased. Moreover, risk factors for mortality by different subgroups were analyzed.

Among the limitations of the study, we highlight the absence of data from biochemical tests of patients in SIVEP-Gripe database, which are relevant in prognostic studies. Another aspect is the notification data of only hospitalized COVID-19 patients, so that we were unable to trace the profile of outpatients. However, our previous study using a dataset from Espírito Santo state [38] can be used for comparative purposes, as it involved COVID-19 outpatients.

## Conclusion

Taken together, our study provides a comprehensive overview of the epidemiological and clinical profile of Brazilian hospitalized COVID-19 patients and the analysis of the risk factors for mortality. We verified that older age and IMV requirement were the most significant risk factors for mortality, besides male gender and the presence of comorbidities. In the absence of enough vaccines or specific therapeutic drugs, prevention is the best strategy to protect people, specially this most vulnerable segment of the population.

The identification of groups at risk for severe COVID-19 is also important to establish priority groups for vaccination, since the initial supply is restricted. We believe this is the first comprehensive study that profiles COVID-19 patients in Brazil and highlights mortality risk factors during hospitalization.

## Supporting information

S1 Table Demographic data of the study population (n = 44,128). (PDF)

S2 Table Clinical data of the study population (n = 44,128). (PDF)

S3 Table Additional information of the study population (n = 44,128). (PDF)

S4 Table Risk factors in fatal outcome using a simple Cox regression model (95% CI). (PDF)

S5 Table Risk factors in fatal outcome using an adjusted Cox regression model (95% CI) for the IMV subgroup. (PDF)

S6 Table Risk factors in fatal outcome using an adjusted Cox regression model (95% CI) for the NIV subgroup. (PDF)

S7 Table Risk factors in fatal outcome using an adjusted Cox regression model (95% CI) for the non ventilation subgroup. (PDF)

S8 Table Risk factors in fatal outcome using an adjusted Cox regression model (95% CI) for the Age <40 subgroup. (PDF)

S9 Table Risk factors in fatal outcome using an adjusted Cox regression model (95% CI) for the Age 40-59 subgroup. (PDF)

S10 Table Risk factors in fatal outcome using an adjusted Cox regression model (95% CI) for the Age 60-79 subgroup. (PDF)

S11 Table Risk factors in fatal outcome using an adjusted Cox regression model (95% CI) for the Age ≥80 subgroup. (PDF)
