# Supplementary material for: On the analysis of mortality risk factors for hospitalized COVID-19 patients: A data-driven study using the major Brazilian database
Source: PLoS One. 2021 Mar 18;16(3):e0248580. doi: 10.1371/journal.pone.0248580 (PMC7971705; doi:10.1371/journal.pone.0248580)
Supplement: S1 Table — (PDF) [file pone.0248580.s001.pdf]

S1 Table: Demographic data of the study population (n=44,128)

	all n(%)	cure n(%)	death n(%)	lr[a](%)
Region	44128 (100.00)	24034 (54.46)	20094 (45.54)	
South	6147 (13.93)	3740 (15.56)	2407 (11.98)	39.16
Southeast	24321 (55.11)	13842 (57.59)	10479 (52.15)	43.09
Midwest	3676 (8.33)	2041 (8.49)	1635 (8.14)	44.48
Northeast	7571 (17.16)	3410 (14.19)	4161 (20.71)	54.96
North	2413 (5.47)	1001 (4.16)	1412 (7.03)	58.52
Age Range[b]				
0-4	233 (0.53)	188 (0.78)	45 (0.22)	19.31
5-9	91 (0.21)	82 (0.34)	9 (0.04)	9.89
10-19	251 (0.57)	203 (0.84)	48 (0.24)	19.12
20-29	942 (2.13)	749 (3.12)	193 (0.96)	20.49
30-39	2661 (6.03)	2098 (8.73)	563 (2.80)	21.16
40-49	4854 (11.00)	3619 (15.06)	1235 (6.15)	25.44
50-59	7881 (17.86)	5264 (21.90)	2617 (13.02)	33.21
60-69	10193 (23.10)	5565 (23.15)	4628 (23.03)	45.40
70-79	9210 (20.87)	3851 (16.02)	5359 (26.67)	58.19
80-89	6139 (13.91)	1984 (8.25)	4155 (20.68)	67.68
90+	1673 (3.79)	431 (1.79)	1242 (6.18)	74.24
Gender				
male	23895 (54.15)	12558 (52.25)	11337 (56.42)	47.45
female	20233 (45.85)	11476 (47.75)	8757 (43.58)	43.28
Race	34505 (100.00)	18307 (53.06)	16198 (46.94)	
white	18329 (53.12)	10304 (56.28)	8025 (49.54)	43.78
black	2337 (6.77)	1213 (6.63)	1124 (6.94)	48.10
asian	467 (1.35)	235 (1.28)	232 (1.43)	49.68
brown	13315 (38.59)	6529 (35.66)	6786 (41.89)	50.97
indigenous	57 (0.17)	26 (0.14)	31 (0.19)	54.39
Education[c]	19376 (100.00)	10750 (55.48)	8626 (44.52)	
illiterate	1386 (7.15)	510 (4.74)	876 (10.16)	63.20
ES-1	5793 (29.90)	2675 (24.88)	3118 (36.15)	53.82
ES-2	3870 (19.97)	2096 (19.50)	1774 (20.57)	45.84
HS	5605 (28.93)	3509 (32.64)	2096 (24.30)	37.40
HE	2549 (13.16)	1822 (16.95)	727 (8.43)	28.52
NA	173 (0.89)	138 (1.28)	35 (0.41)	20.23

[a]lethality rate

[b]in years

[c]ES-1= Elementary School 1; ES-2= Elementary School 2; HS= High School; HE= Higher Education, NA= Not Applicable (age<7)
