# Supplementary material for: On the analysis of mortality risk factors for hospitalized COVID-19 patients: A data-driven study using the major Brazilian database
Source: PLoS One. 2021 Mar 18;16(3):e0248580. doi: 10.1371/journal.pone.0248580 (PMC7971705; doi:10.1371/journal.pone.0248580)
Supplement: S2 Table — (PDF) [file pone.0248580.s002.pdf]

S2 Table: Clinical data of the study population (n=44,128)

	all n(%)	cure n(%)	death n(%)
**Symptom**			
Fever (n=44128)	29824 (67.59)	16800 (56.33)	13024 (43.67)
Cough (n=44128)	33008 (74.80)	18762 (56.84)	14246 (43.16)
Sore Throat (n=44128)	7601 (17.22)	4522 (59.49)	3079 (40.51)
Dispnoea (n=44128)	33053 (74.90)	16675 (50.45)	16378 (49.55)
Respiratory Distress (n=44128)	27863 (63.14)	13518 (48.52)	14345 (51.48)
SP O2 $< 95\%$[a] (n=44128)	29358 (66.53)	13882 (47.29)	15476 (52.71)
Diarrhea (n=44128)	6338 (14.36)	3940 (62.16)	2398 (37.84)
Vomit (n=44128)	3766 (8.53)	2273 (60.36)	1493 (39.64)
Other (n=44128)	17488 (39.63)	10889 (62.27)	6599 (37.73)
**Comorbidity**			
Cardiac disease (n=44128)	22957 (52.02)	11827 (51.52)	11130 (48.48)
Hematological disease (n=44128)	650 (1.47)	305 (46.92)	345 (53.08)
Down's syndrome (n=44128)	203 (0.46)	108 (53.20)	95 (46.80)
Liver disease (n=44128)	733 (1.66)	298 (40.65)	435 (59.35)
Asthma (n=44128)	2118 (4.80)	1425 (67.28)	693 (32.72)
Diabetes (n=44128)	17573 (39.82)	8825 (50.22)	8748 (49.78)
Neuropathy (n=44128)	2866 (6.49)	1051 (36.67)	1815 (63.33)
Pneumopathy (n=44128)	2788 (6.32)	1101 (39.49)	1687 (60.51)
Immunodepression (n=44128)	2343 (5.31)	1046 (44.64)	1297 (55.36)
Kidney disease (n=44128)	3227 (7.31)	1176 (36.44)	2051 (63.56)
Obesity (n=44128)	3633 (8.23)	2119 (58.33)	1514 (41.67)
Other (n=44128)	20081 (45.51)	10548 (52.53)	9533 (47.47)

[a] oxygen saturation
