# Supplementary material for: On the analysis of mortality risk factors for hospitalized COVID-19 patients: A data-driven study using the major Brazilian database
Source: PLoS One. 2021 Mar 18;16(3):e0248580. doi: 10.1371/journal.pone.0248580 (PMC7971705; doi:10.1371/journal.pone.0248580)
Supplement: S3 Table — (PDF) [file pone.0248580.s003.pdf]

S3 Table: Additional information of the study population (n=44,128)

	all n(%)	cure n(%)	death n(%)
Flu Vaccine	8234 (18.66)	4729 (57.43)	3505 (42.57)
Flu Antiviral	14732 (33.38)	8161 (55.40)	6571 (44.60)
ICU admission	18590 (42.13)	6222 (33.47)	12368 (66.53)
Ventilation	32940 (74.65)	15395 (46.74)	17545 (53.26)
IMV[a]	11747 (26.62)	1798 (15.31)	9949 (84.69)
NIV[b]	21193 (48.03)	13597 (64.16)	7596 (35.84)

[a]Invasive Mechanical Ventilation

[b]Non Invasive Ventilation
