# Supplementary material for: On the analysis of mortality risk factors for hospitalized COVID-19 patients: A data-driven study using the major Brazilian database
Source: PLoS One. 2021 Mar 18;16(3):e0248580. doi: 10.1371/journal.pone.0248580 (PMC7971705; doi:10.1371/journal.pone.0248580)
Supplement: S4 Table — (PDF) [file pone.0248580.s004.pdf]

S4 Table: Risk factors in fatal outcome using an unadjusted Cox regression model (95% CI)

Variable[a]	HR	CI 95%	p-value
Male	1.12	(1.09-1.15)	<0.001
Age 40-60	0.50	(0.48-0.51)	<0.001
Age 60-80	1.34	(1.30-1.37)	<0.001
Age >80	2.27	(2.20-2.34)	<0.001
Fever	0.83	(0.81-0.86)	<0.001
Cough	0.75	(0.73-0.77)	<0.001
Sore Throat	0.83	(0.80-0.87)	<0.001
Dispnoea	1.69	(1.63-1.75)	<0.001
Respiratory Distress	1.68	(1.63-1.73)	<0.001
SP O2 <95%	2.01	(1.94-2.08)	<0.001
Diarrhea	0.75	(0.72-0.78)	<0.001
Vomit	0.82	(0.78-0.86)	<0.001
Other symptom	0.67	(0.65-0.69)	<0.001
Cardiac disease	1.19	(1.16-1.23)	<0.001
Hematological disease	1.21	(1.09-1.35)	<0.001
Down's syndrome	1.05	(0.85-1.28)	0.669
Liver disease	1.47	(1.33-1.61)	<0.001
Asthma	0.64	(0.59-0.69)	<0.001
Diabetes	1.23	(1.20-1.27)	<0.001
Neuropathy	1.69	(1.61-1.77)	<0.001
Pneumopathy	1.54	(1.46-1.62)	<0.001
Immunodepression	1.30	(1.23-1.38)	<0.001
Kidney disease	1.65	(1.57-1.72)	<0.001
Obesity	0.87	(0.82-0.92)	<0.001
Other comorbidity	1.10	(1.07-1.13)	<0.001
Flu Vaccine	0.89	(0.86-0.92)	<0.001
Flu Antiviral	0.94	(0.92-0.97)	<0.001
ICU admission	2.74	(2.66-2.82)	<0.001
IMV	4.30	(4.18-4.42)	<0.001
NIV	0.58	(0.57-0.60)	<0.001

[a]see S2 Table; n=44,128 patients with full symptom/comorbidity information
