# Supplementary material for: On the analysis of mortality risk factors for hospitalized COVID-19 patients: A data-driven study using the major Brazilian database
Source: PLoS One. 2021 Mar 18;16(3):e0248580. doi: 10.1371/journal.pone.0248580 (PMC7971705; doi:10.1371/journal.pone.0248580)
Supplement: S5 Table — (PDF) [file pone.0248580.s005.pdf]

S5 Table: Risk factors in fatal outcome using an adjusted Cox regression model (95% CI) for the IMV subgroup

Variable	HR	CI 95%	p value
Age 40-60	1.14	(1.04-1.25)	0.008
Age 60-80	1.53	(1.40-1.68)	<0.001
Age >80	2.05	(1.86-2.26)	<0.001
Fever	0.90	(0.87-0.94)	<0.001
Cough	0.89	(0.85-0.93)	<0.001
Respiratory Distress	1.17	(1.11-1.23)	<0.001
SP O2 <95%	1.14	(1.08-1.21)	<0.001
Diarrhea	0.91	(0.85-0.97)	<0.005
Other symptom	0.91	(0.88-0.95)	<0.001
Liver disease	1.18	(1.03-1.35)	0.018
Asthma	0.82	(0.74-0.91)	<0.001
Diabetes	1.11	(1.06-1.15)	<0.001
Pneumopathy	1.08	(1.01-1.16)	0.025
Immunodepression	1.09	(1.00-1.18)	0.043
Kidney disease	1.15	(1.08-1.22)	<0.001
Obesity	0.93	(0.87-1.00)	0.035
Flu Vaccine	0.89	(0.84-0.94)	<0.001
Flu Antiviral	0.90	(0.86-0.94)	<0.001
ICU admission	0.80	(0.76-0.85)	<0.001
