# Supplementary material for: On the analysis of mortality risk factors for hospitalized COVID-19 patients: A data-driven study using the major Brazilian database
Source: PLoS One. 2021 Mar 18;16(3):e0248580. doi: 10.1371/journal.pone.0248580 (PMC7971705; doi:10.1371/journal.pone.0248580)
Supplement: S6 Table — (PDF) [file pone.0248580.s006.pdf]

S6 Table: Risk factors in fatal outcome using an adjusted Cox regression model (95% CI) for the NIV subgroup

Variable	HR	CI 95%	*p* value
Male	1.17	(1.12-1.23)	<0.001
Age 40-60	1.61	(1.39-1.86)	<0.001
Age 60-80	3.47	(3.02-4.00)	<0.001
Age >80	6.64	(5.75-7.66)	<0.001
Fever	0.94	(0.90-0.99)	0.016
Cough	0.82	(0.78-0.87)	<0.001
Dispnoea	1.27	(1.19-1.35)	<0.001
Respiratory Distress	1.27	(1.20-1.34)	<0.001
SP O2 <95%	1.28	(1.20-1.35)	<0.001
Diarrhea	0.91	(0.85-0.98)	0.007
Other symptom	0.73	(0.69-0.76)	<0.001
Cardiac disease	0.90	(0.86-0.95)	<0.001
Hematological disease	1.26	(1.06-1.50)	0.010
Liver disease	1.36	(1.15-1.60)	<0.001
Asthma	0.72	(0.63-0.82)	<0.001
Diabetes	1.10	(1.05-1.15)	<0.001
Neuropathy	1.35	(1.25-1.45)	<0.001
Immunodepression	1.48	(1.34-1.62)	<0.001
Kidney disease	1.38	(1.27-1.49)	<0.001
Other comorbidity	1.09	(1.04-1.15)	<0.001
Flu Antiviral	0.90	(0.85-0.94)	<0.001
ICU admission	1.43	(1.37-1.50)	<0.001
