# Supplementary material for: On the analysis of mortality risk factors for hospitalized COVID-19 patients: A data-driven study using the major Brazilian database
Source: PLoS One. 2021 Mar 18;16(3):e0248580. doi: 10.1371/journal.pone.0248580 (PMC7971705; doi:10.1371/journal.pone.0248580)
Supplement: S7 Table — (PDF) [file pone.0248580.s007.pdf]

S7 Table: Risk factors in fatal outcome using an adjusted Cox regression model (95% CI) for the non ventilation subgroup

Variable	HR	CI 95%	p value
Male	1.19	(1.10-1.29)	<0.001
Age 40-60	1.86	(1.51-2.31)	<0.001
Age 60-80	4.17	(3.41-5.11)	<0.001
Age >80	8.22	(6.68-10.12)	<0.001
Cough	0.80	(0.73-0.87)	<0.001
Dispnoea	1.31	(1.19-1.43)	<0.001
Respiratory Distress	1.23	(1.13-1.34)	<0.001
SP O2 <95%	1.55	(1.42-1.68)	<0.001
Diarrhea	0.78	(0.69-0.87)	<0.001
Other symptom	0.60	(0.55-0.66)	<0.001
Hematological disease	1.33	(1.02-1.72)	0.033
Liver disease	1.56	(1.21-2.00)	<0.001
Diabetes	1.10	(1.01-1.19)	0.025
Neuropathy	1.46	(1.28-1.67)	<0.001
Immunodepression	1.61	(1.39-1.87)	<0.001
Kidney disease	1.47	(1.29-1.68)	<0.001
Other comorbidity	1.22	(1.13-1.33)	<0.001
Flu Antiviral	0.80	(0.73-0.88)	<0.001
ICU admission	1.57	(1.44-1.72)	<0.001
