# Supplementary material for: On the analysis of mortality risk factors for hospitalized COVID-19 patients: A data-driven study using the major Brazilian database
Source: PLoS One. 2021 Mar 18;16(3):e0248580. doi: 10.1371/journal.pone.0248580 (PMC7971705; doi:10.1371/journal.pone.0248580)
Supplement: S8 Table — (PDF) [file pone.0248580.s008.pdf]

S8 Table: Risk factors in fatal outcome using an adjusted Cox regression model (95% CI) for the Age <40 subgroup

Variable	HR	CI 95%	*p* value
Fever	0.81	(0.69-0.94)	0.006
Respiratory Distress	1.18	(1.00-1.40)	0.047
SP O2 <95%	1.52	(1.27-1.81)	<0.001
Other symptom	0.76	(0.66-0.88)	<0.001
Down's syndrome	1.75	(1.16-2.64)	0.008
Asthma	0.62	(0.47-0.83)	<0.005
Diabetes	1.38	(1.17-1.61)	<0.001
Immunodepression	1.71	(1.42-2.07)	<0.001
Kidney disease	1.34	(1.06-1.69)	0.013
Other comorbidity	1.24	(1.08-1.43)	<0.005
Flu Vaccine	0.70	(0.54-0.91)	0.007
IMV	11.57	(9.22-14.53)	<0.001
NIV	1.68	(1.32-2.15)	<0.001
