# Supplementary material for: On the analysis of mortality risk factors for hospitalized COVID-19 patients: A data-driven study using the major Brazilian database
Source: PLoS One. 2021 Mar 18;16(3):e0248580. doi: 10.1371/journal.pone.0248580 (PMC7971705; doi:10.1371/journal.pone.0248580)
Supplement: S9 Table — (PDF) [file pone.0248580.s009.pdf]

S9 Table: Risk factors in fatal outcome using an adjusted Cox regression model (95% CI) for the Age 40-59 subgroup

Variable	HR	CI 95%	p value
Male	1.09	(1.02-1.16)	0.014
Fever	0.89	(0.82-0.95)	<0.001
Cough	0.87	(0.80-0.94)	<0.001
Dispnoea	1.13	(1.03-1.24)	0.009
Respiratory Distress	1.19	(1.10-1.29)	<0.001
SP O2 <95%	1.25	(1.15-1.36)	<0.001
Other symptom	0.75	(0.70-0.81)	<0.001
Liver disease	1.53	(1.28-1.83)	<0.001
Asthma	0.80	(0.69-0.93)	<0.005
Diabetes	1.25	(1.17-1.33)	<0.001
Neuropathy	1.36	(1.16-1.60)	<0.001
Pneumopathy	1.22	(1.06-1.41)	0.006
Immunodepression	1.40	(1.24-1.57)	<0.001
Kidney disease	1.46	(1.32-1.62)	<0.001
Other comorbidity	1.12	(1.05-1.19)	<0.001
Flu Antiviral	0.83	(0.78-0.89)	<0.001
ICU admission	1.44	(1.32-1.57)	<0.001
IMV	5.91	(5.23-6.68)	<0.001
NIV	1.37	(1.23-1.53)	<0.001
