# Supplementary material for: On the analysis of mortality risk factors for hospitalized COVID-19 patients: A data-driven study using the major Brazilian database
Source: PLoS One. 2021 Mar 18;16(3):e0248580. doi: 10.1371/journal.pone.0248580 (PMC7971705; doi:10.1371/journal.pone.0248580)
Supplement: S10 Table — (PDF) [file pone.0248580.s010.pdf]

S10 Table: Risk factors in fatal outcome using an adjusted Cox regression model (95% CI) for the Age 60-79 subgroup

Variable	HR	CI 95%	p value
Male	1.13	(1.08-1.17)	<0.001
Fever	0.92	(0.88-0.96)	<0.001
Cough	0.84	(0.80-0.88)	<0.001
Dispnoea	1.20	(1.14-1.27)	<0.001
Respiratory Distress	1.20	(1.14-1.25)	<0.001
SP O2 <95%	1.23	(1.17-1.29)	<0.001
Diarrhea	0.87	(0.82-0.92)	<0.001
Other symptom	0.79	(0.76-0.83)	<0.001
Cardiac disease	0.95	(0.91-0.99)	0.018
Liver disease	1.21	(1.06-1.39)	0.006
Asthma	0.87	(0.78-0.98)	0.021
Diabetes	1.10	(1.05-1.14)	<0.001
Neuropathy	1.23	(1.14-1.33)	<0.001
Immunodepression	1.21	(1.12-1.32)	<0.001
Kidney disease	1.23	(1.16-1.32)	<0.001
Other comorbidity	1.05	(1.01-1.10)	0.012
Flu Antiviral	0.91	(0.87-0.95)	<0.001
ICU admission	1.29	(1.23-1.36)	<0.001
IMV	3.69	(3.42-3.97)	<0.001
NIV	1.31	(1.23-1.40)	<0.001
