# Supplementary material for: On the analysis of mortality risk factors for hospitalized COVID-19 patients: A data-driven study using the major Brazilian database
Source: PLoS One. 2021 Mar 18;16(3):e0248580. doi: 10.1371/journal.pone.0248580 (PMC7971705; doi:10.1371/journal.pone.0248580)
Supplement: S11 Table — (PDF) [file pone.0248580.s011.pdf]

S11 Table: Risk factors in fatal outcome using an adjusted Cox regression model (95% CI) for the Age ≥80 subgroup

Variable	HR	CI 95%	*p* value
Male	1.10	(1.05-1.17)	<0.001
Cough	0.86	(0.82-0.91)	<0.001
Dispnoea	1.19	(1.11-1.28)	<0.001
Respiratory Distress	1.22	(1.14-1.30)	<0.001
SP O2 <95%	1.25	(1.17-1.34)	<0.001
Vomit	0.88	(0.79-0.99)	0.029
Other symptom	0.82	(0.77-0.87)	<0.001
Cardiac disease	0.91	(0.86-0.96)	<0.001
Liver disease	1.31	(1.03-1.68)	0.031
Asthma	0.84	(0.72-0.98)	0.025
Neuropathy	1.15	(1.07-1.24)	<0.001
Kidney disease	1.12	(1.02-1.23)	0.014
Flu Antiviral	0.87	(0.82-0.92)	<0.001
ICU admission	1.08	(1.02-1.16)	0.015
IMV	2.50	(2.27-2.76)	<0.001
NIV	1.28	(1.18-1.40)	<0.001
